# Supplementary material for: Superhydrophobic hemostatic nanofiber composites for fast clotting and minimal adhesion
Source: Nat Commun. 2019 Dec 5;10:5562. doi: 10.1038/s41467-019-13512-8 (PMC6895059; doi:10.1038/s41467-019-13512-8)
Supplement: Supplementary file 1 — Supplementary Information [file 41467_2019_13512_MOESM1_ESM.pdf]

Supplementary Information

Superhydrophobic Hemostatic Nanofiber Composites for Fast Clotting and Minimal Adhesion

Zhe Li[1,2], Athanasios Milionis[4], Yu Zheng[2], Marcus Yee[2], Lukas Codispoti[4], Freddie Tan[3], Dimos Poulikakos[4*], Choon Hwai Yap[2*]

[1] Guangdong Provincial Key Laboratory of Sensor Technology and Biomedical Instrument, School of Biomedical Engineering, Sun Yat-Sen University, Guangzhou, P. R. China, 510006.

[2] Department of Biomedical Engineering, National University of Singapore, Singapore, 117583.

[3] Department of Chemical and Biomolecular Engineering, National University of Singapore, Singapore, 117585.

[4] Laboratory of Thermodynamics in Emerging Technologies, Department of Mechanical and Process Engineering, ETH Zurich, 8092 Zurich, Switzerland.

Corresponding authors:

1. Choon Hwai Yap. Email: bieyapc@nus.edu.sg

2. Dimos Poulikakos. Email: dpoulikakos@ethz.ch

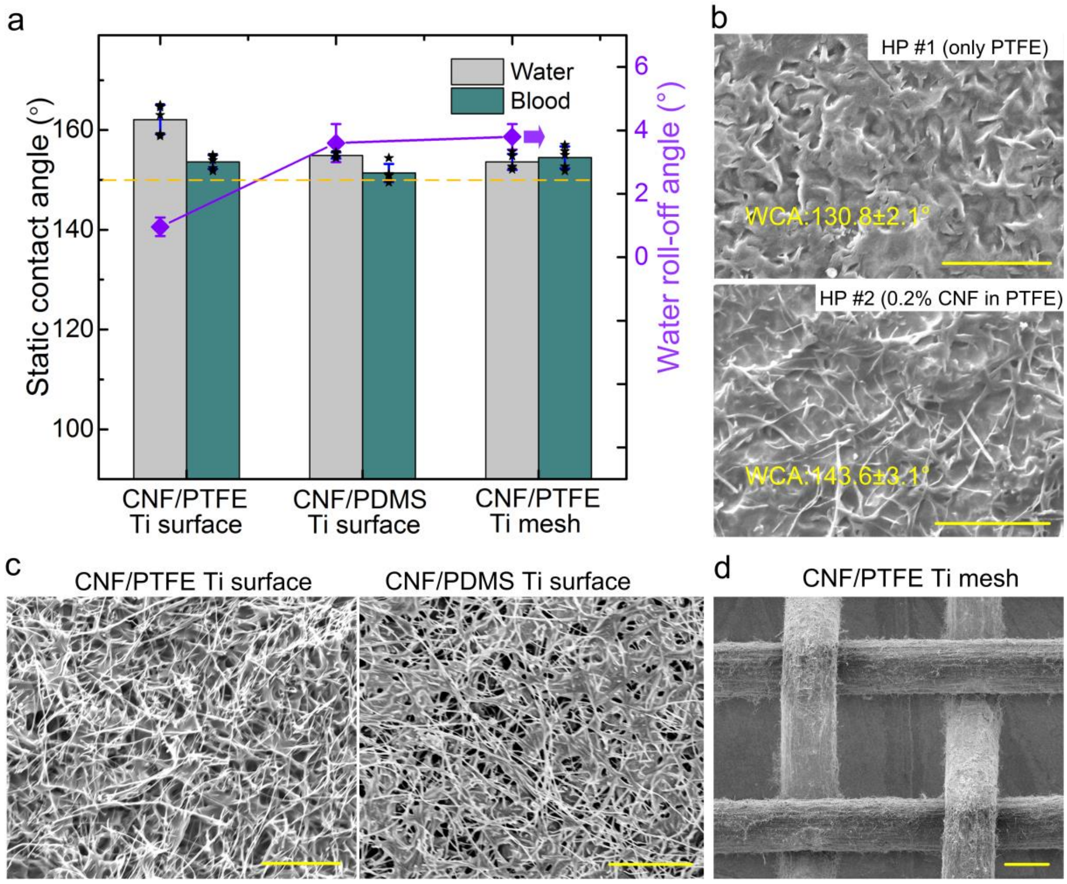

Supplementary Figure 1. Hydrophobicity and surface morphology of different CNF nano-composite coatings. (a) Water contact angle & roll-off angle, and blood contact angle on different superhydrophobic surfaces; data are shown as mean ± s.d., and the error bar represents s.d. (n = 5); individual data points for water and blood contact angles are represented by black stars. (b) SEM images of the hydrophobic surfaces HP #1 and HP #2; HP #1 refers to a hydrophobic Ti surface coated with only PTFE that has a water contact angle of 130.8±2.1°; HP # 2 refers to a Ti surface coated with 0.2 wt% CNF in PTFE that has a water contact angle of 143.6±3.1°. (c) SEM images of superhydrophobic nano-composite CNF surfaces; the CNF/PTFE surface had a surface morphology similar to the CNF/PDMS surface. (d) The superhydrophobic CNF/PTTE Ti mesh sample. On the superhydrophobic CNF/PTFE and CNF/PDMS Ti surfaces, blood had a smaller contact angle than water (but still larger than 150°) due to the lower surface tension of blood. On the superhydrophobic CNF/PTFE Ti mesh surface, the contact angle could not be accurately measured due to the special mesh topography. On the hydrophobic surfaces (HP #1 and HP #2), the reduced surface roughness lead to a smaller water contact angle.. Scale bars are 15 μm in b and c, and 100 μm in d.

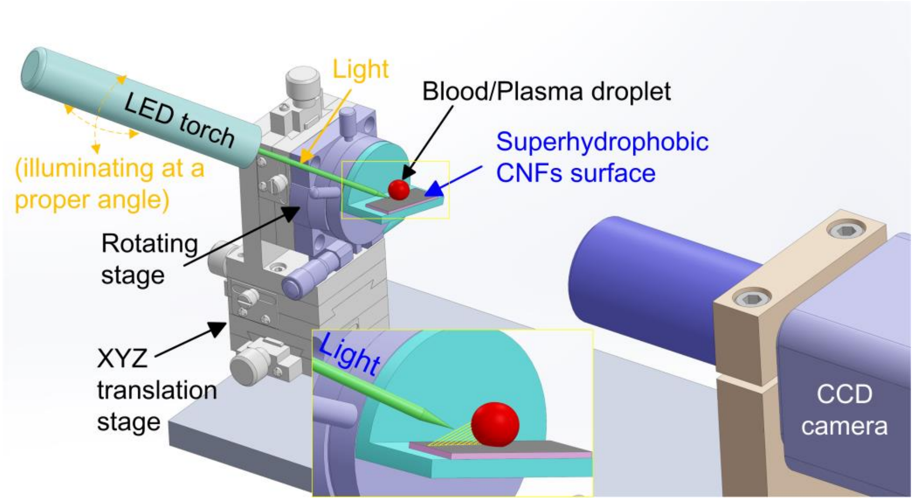

Supplementary Figure 2. Experimental setup. This setup is used to visualize fibrin fibers generating at the receding side of the blood/plasma droplet sliding down a superhydrophobic CNF surface. In order to visualize fibrin fibers during blood or PPP sliding, backlighting in a typical goniometer was avoided due to over-exposure; a light source was projected at a proper angle from the side to make fibrin fibers visible.

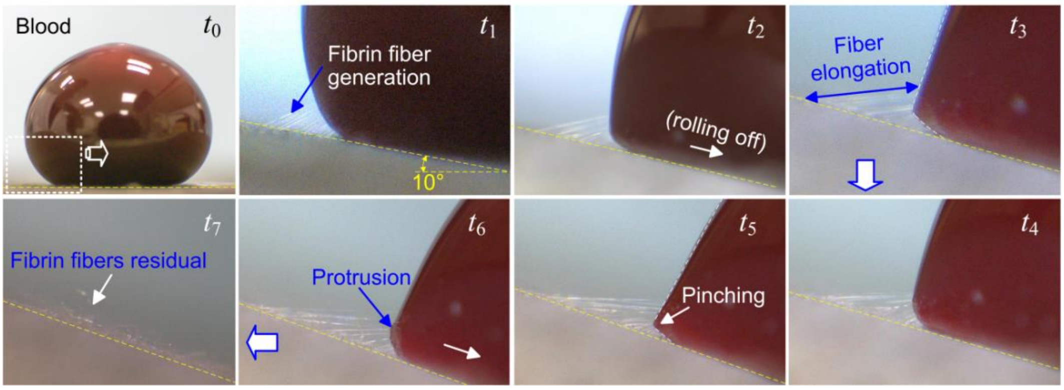

Supplementary Figure 3. Fibrin fiber generation. Fibrin fibers are generating at the receding side of a 20 µl EDTA blood droplet sliding down a superhydrophobic CNF/PTFE Ti surface (Supplementary Movie 2). Similar fibrin fiber generation phenomenon was also observed for EDTA or citrated blood/PPP droplets sliding on the CNF/PTFE surface.

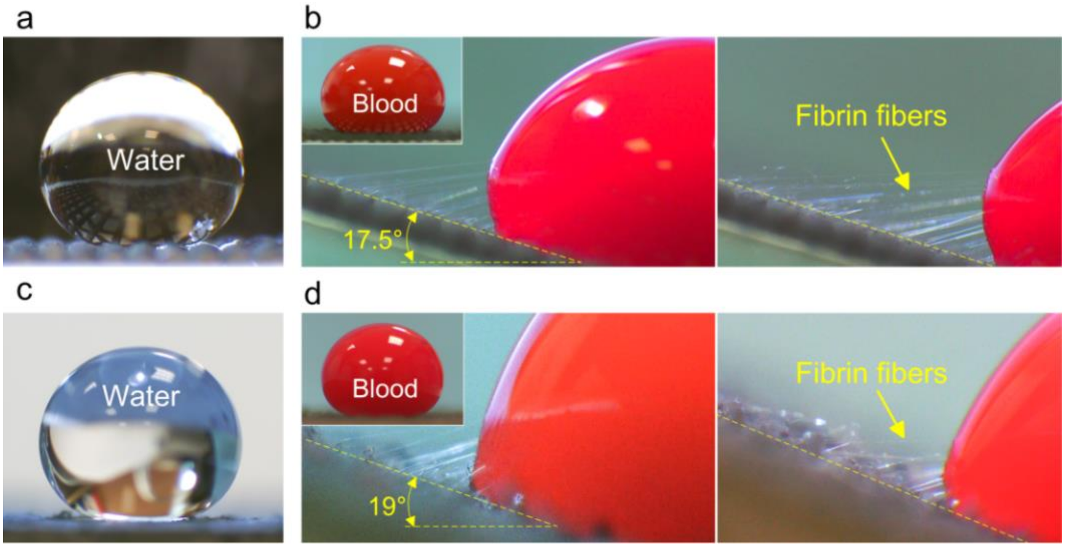

Supplementary Figure 4. Fibrin fiber generation on the CNF/PTFE Ti mesh and the CNF/PDMS surface. (a) A 20 μl water droplet sitting on the superhydrophobic CNF/PTFE Ti mesh. (b) Fibrin fibers generating at the receding side of a 40 μl citrated blood droplet on the superhydrophobic CNF Ti mesh (Supplementary Movie 3). (c) A 20 μl water droplet sitting on the superhydrophobic CNF/PDMS Ti surface. (d) Fibrin fibers generating at the receding side of a 20 μl citrated blood droplet on the superhydrophobic CNF/PDMS Ti surface (Supplementary Movie 5). Fibrin fibers were also observed for PPP with EDTA on this CNF/PDMS Ti surface (Supplementary Movie 4). Similar fibrin fiber generation phenomenon was observed for all EDTA or citrated blood/PPP droplets sliding on the CNF/PTFE Ti mesh and the CNF/PDMS Ti surfaces.

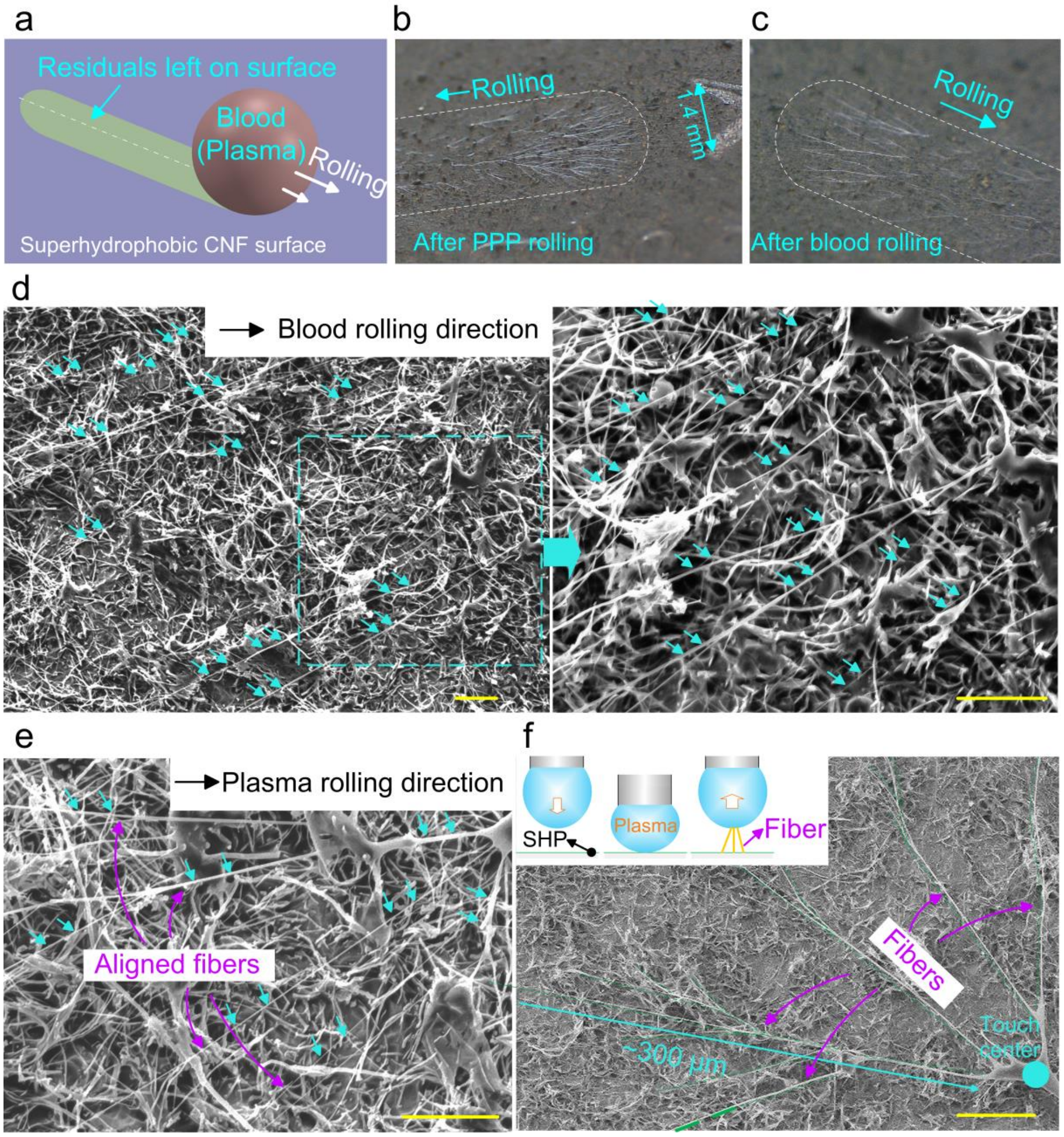

Supplementary Figure 5. Fibrin fibers generation on the superhydrophobic CNF/PTFE Ti surface. (a) Illustration of blood/plasma sliding. (b-c) Optical images showing fibrin fibers left on the surface after PPP or blood sliding, and visible fibrin fiber footprints were left on the CNF surface after sliding tests. (d) SEM images of the CNF surface after blood sliding; long, straight and aligned fibrin fibers can be observed on top of random CNFs; arrows are used to highlight the aligned fibrin fibers. (e) SEM image of the long, straight and aligned fibrin fibers left on the CNF surface after the PPP droplet rolled down the superhydrophobic CNF surface. (f) SEM image of the long and straight fibrin fibers left on the CNF surface after the PPP droplet touch-lift test; SHP in the inset means the superhydrophobic CNF surface. Scale bars are 10 μm in d and e, and 50 μm in f.

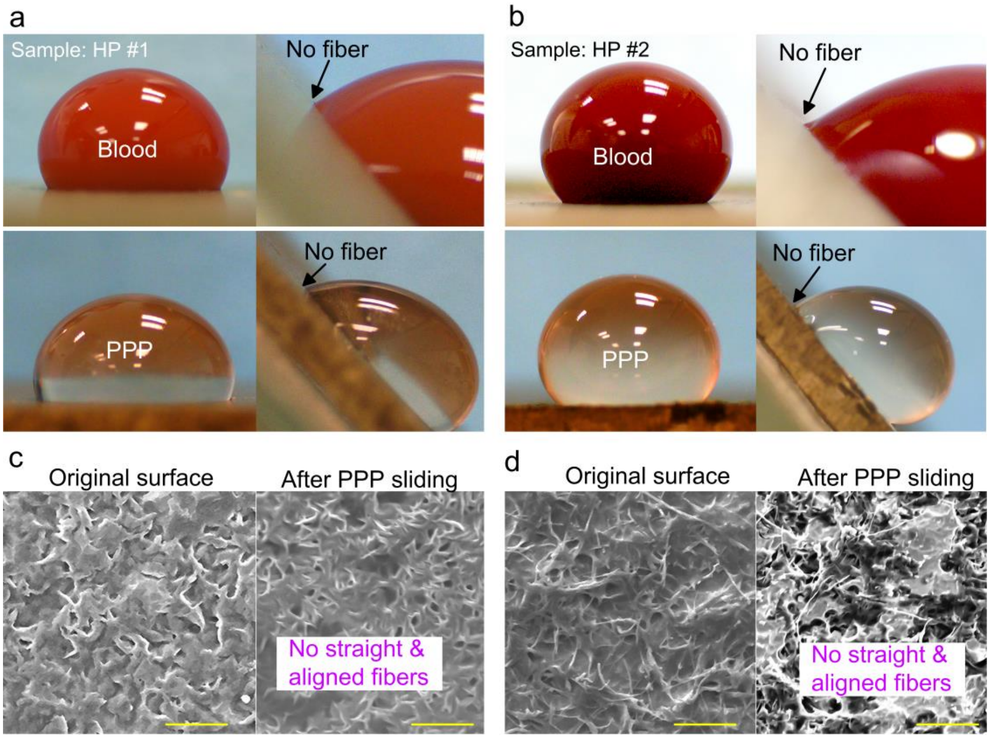

Supplementary Figure 6. No fibrin fiber generating on the hydrophobic CNF surfaces. (a) No fiber observed for 20 µl EDTA blood or PPP droplets on the HP #1 sample (spray-coated with only PTFE). (b) No fiber observed for 20 µl blood or PPP droplets on the HP #2 sample (spray-coated with 0.2 wt% CNF in PTFE). (c) SEM images of the HP #1 surface before and after PPP sliding, showing no straight fibrin fibers on the surface; instead, the surface became bio-fouled by plasma; similar results were observed on this surface after blood sliding. (d) SEM images of the HP #2 surface before and after PPP sliding, showing no straight fibrin fibers on the surface too; the surface also became bio-fouled by plasma; similar results were observed on this surface after blood sliding. Scale bars are 10 µm in c and d.

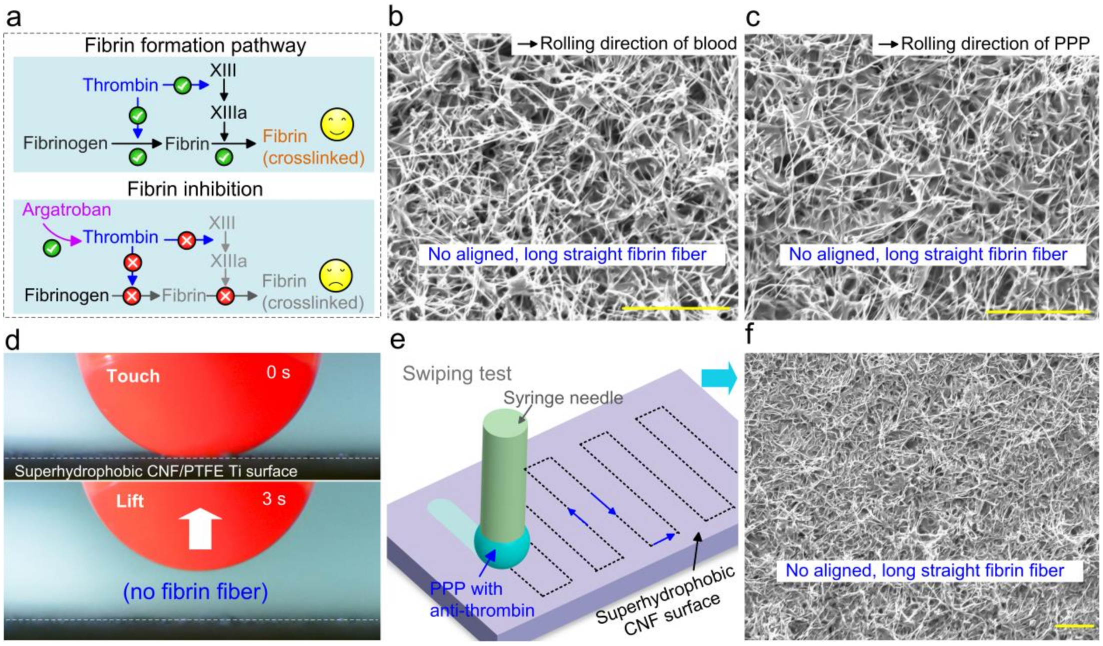

Supplementary Figure 7. No fibrin fiber generation for blood/PPP with anti-thrombin. (a) Fibrin inhibition by anti-thrombin, argatroban. (b-c) SEM images of the CNF surface after sliding by anti-thrombin blood and PPP, respectively, showing no aligned/straight fibrin fiber. (d) No fibrin fiber was detected when a blood drop with anti-thrombin came into contact and was subsequently lifted from the superhydrophobic CNF surface. (e) Swiping the CNF surface by PPP with anti-thrombin generated no fibrin fibers. (f) SEM image of the CNF surface after PPP swiping test, showing no aligned/straight fibrin fiber. Scale bars are 20 μm in b, c and f.

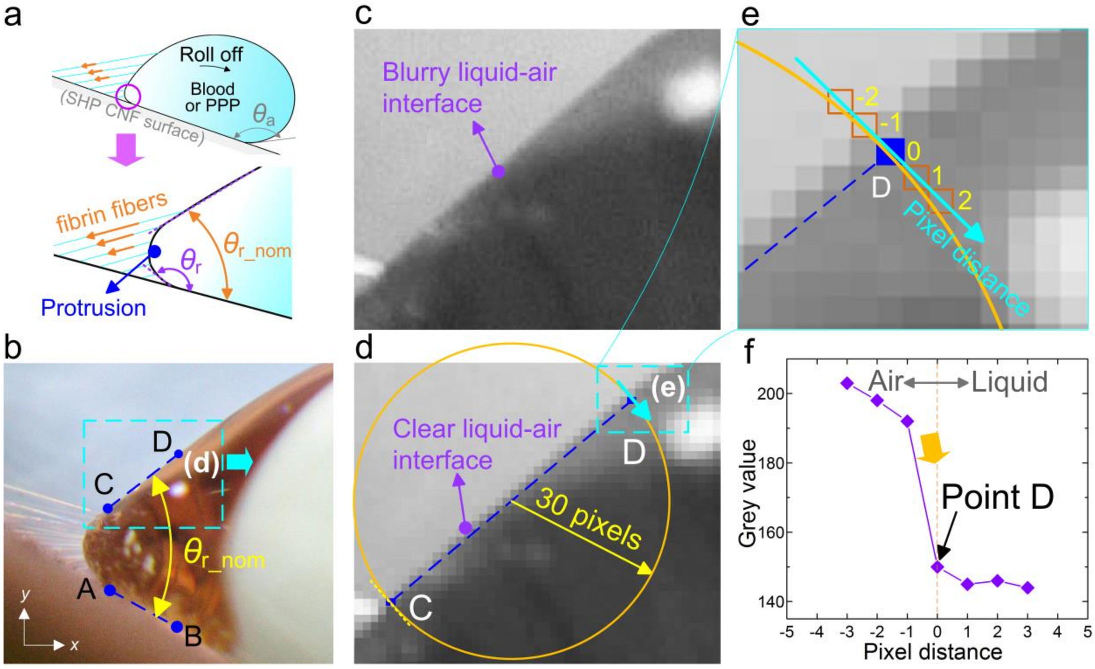

Supplementary Figure 8. Nominal receding angle. (a) Due to the droplet protrusion, the nominal receding angle θ_{r_nom} is different from the actual receding angle θ_r for blood or PPP droplets on the superhydrophobic CNF surfaces. (b) θ_{r_nom}, measured at the moment before catastrophic fibrin fiber fracture and droplet rolling down, is the angle between line AB and line CD; AB is at the droplet-substrate interface; CD is at the straight portion of the droplet-air interface above the droplet protrusion. (c) The liquid-air interface is blurry on the high-resolution (1920×1080 pixels) black and white image. (d) The liquid-air or liquid-substrate interface was smoothed via pixel intensity averaging and reducing pixel resolution image to 480×270 pixels. This made it easier to localize lines AB and CD. A reference circle, with a radius of 30 pixels, was drawn to intersect the straight portion of the liquid-air interface at two points C and D. (e-f) The position of D was further confirmed at the point on the circle where the pixel grey value had an abrupt change; pixels outside the liquid phase are labeled with "-" in (e). The position of C was confirmed similarly. Line AB on the liquid-solid interface was determined using the same method in d-f. The aforementioned procedures were performed manually, and had sufficient accuracy, with errors being in the order of a pixel width, which translated to an angle error of about 0.95° (equaled to arctan(1/60)).

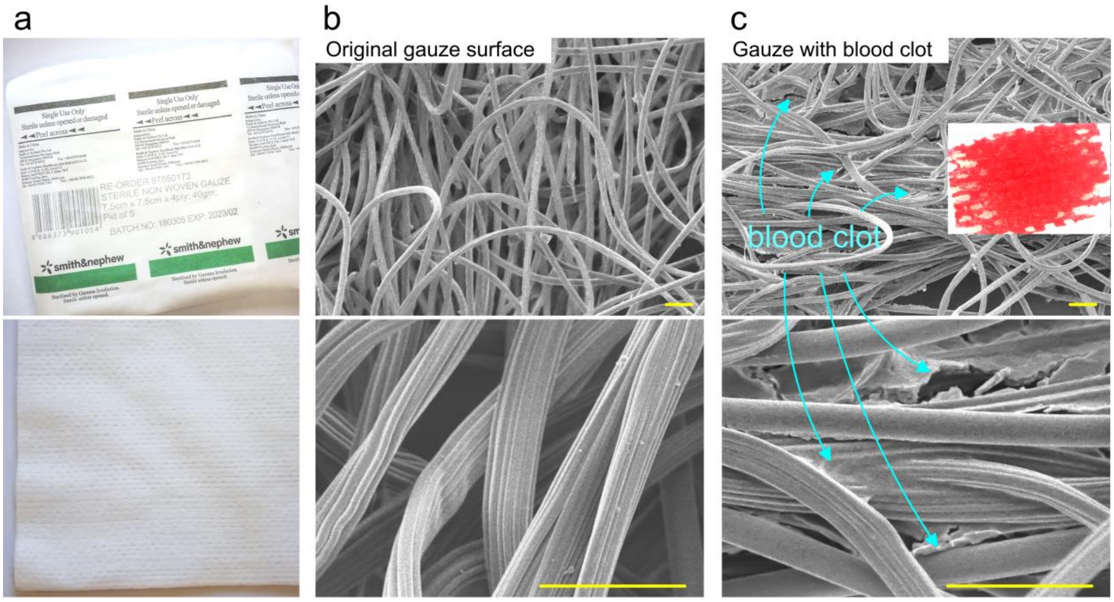

Supplementary Figure 9. The normal cotton gauze (Smith & Nephew Pte Ltd). (a) Optical images of the uncoated gauze. (b) SEM images of the uncoated gauze. (c) SEM images of the gauze with clot, the clot and gauze being a solid piece. Scale bars are 50 µm in b and c.

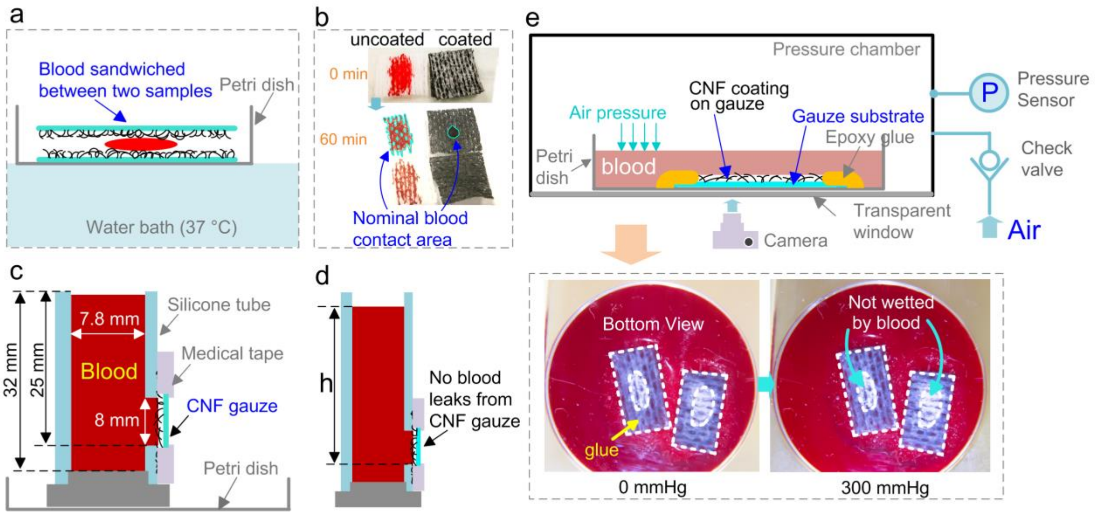

Supplementary Figure 10. *In vitro* clotting tests. (a) Clotting test by sandwiching 20 µl blood between two gauze samples (size: 15 mm by 15 mm); for the superhydrophobic CNF gauze, blood was contacting the surface coated with CNF. (b) Nominal blood contact area of 20 µl blood on the uncoated white cotton gauze (Smith & Nephew Pte Ltd; Supplementary Figure 9a) and a superhydrophobic CNF-coated gauze. The blood became sufficiently coagulated after 60 minutes, making it easier to separate the two gauzes and to measure the blood contact area; the blood contact contour is highlighted by the green line. (c) Clotting without blood loss; the superhydrophobic CNF gauze was used to seal an opening (8 mm by 5 mm) on the silicone tube by medical tape, which was subsequently filled with blood, initiating coagulation by adding $CaCl_2$. (d) Measurement of the maximum hydrostatic pressure that one layer of the CNF gauze (without an impervious membrane) can withstand before blood leakage; the maximum blood column height *h* without blood leakage was used to calculate the blood leakage-free pressure. (e) Non-wetting of the CNF gauze by blood under high pressure; the CNF gauze, the periphery of which (not the central region) was glued onto the petri dish, was immersed in the citrated blood, in a pressure chamber with the CNF surface contacting blood; the petri dish acted as an impervious membrane to retain the air plastron across the CNF/blood interface; the hydrostatic pressure exerted onto the CNF surface in blood was controlled by pumping air into the pressure chamber, monitoring the air pressure by a pressure sensor; a camera projecting upward was used to detect whether the central part of the CNF gauze was wetted by blood at a given pressure; in comparison, it was experimentally observed that the normal gauze instantly got wetted by blood at ambient pressure. The CNF coating was in contact with blood in these tests.

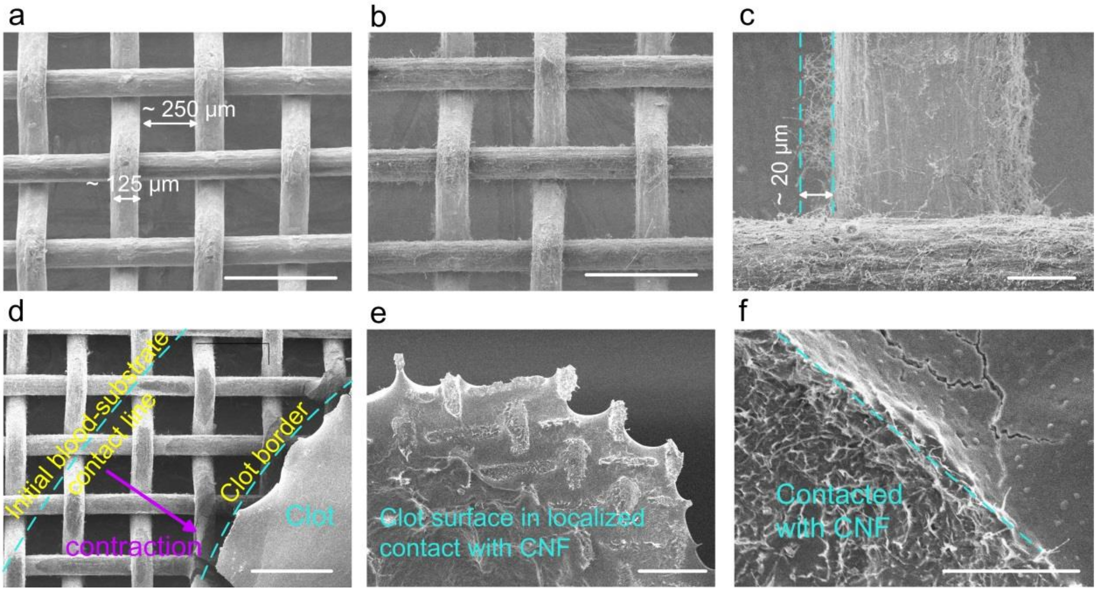

Supplementary Figure 11. Clot self-detachment on the stiff CNF/PTFE Ti mesh. (a) SEM of the pristine Ti mesh #60 before CNF coating, with a nominal aperture of about 250 μm and a wire diameter of about 125 μm. (b-c) SEM images of the Ti mesh after coating with superhydrophobic CNF (thickness of the CNF layer was approximately 20 μm). (d) Clot contraction on the CNF Ti mesh; the clot border contracted inwards from the initial blood-substrate contact line. (e) A clot self-detached from the CNF Ti mesh, showing localized contact between blood and the Ti mesh. (f) The hairy area on the clot, which was in contact with CNF. Scale bars are 500 μm in a, b, d and e, and 50 μm in c and f.

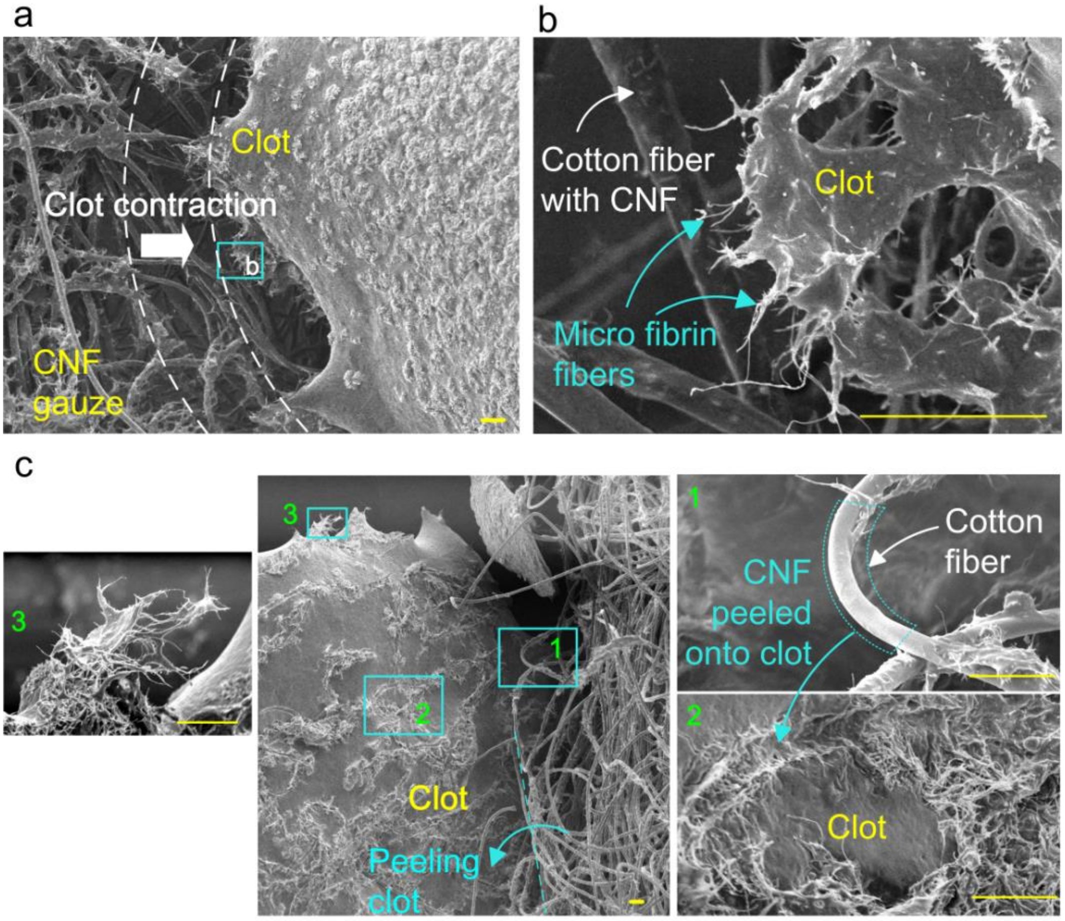

Supplementary Figure 12. Facile clot detachment. (a) SEM image showing the aftermath of the clot contraction on the CNF gauze. (b) Some micro fibrin fibers remain adhered on the CNF-coated cotton fiber after clot shrinkage. (c) SEM images of the clot surface in contact with the CNF gauze after clot detachment, showing localized contact between blood and the CNF gauze; c-1 and c-2 show CNFs transferred onto clot after clot detachment, resulting in a smooth cotton fiber, and a hairy clot surface; c-3 shows micro fibrin fibers initially attached onto CNFs. Scale bars are 50 μm in a, b and c.

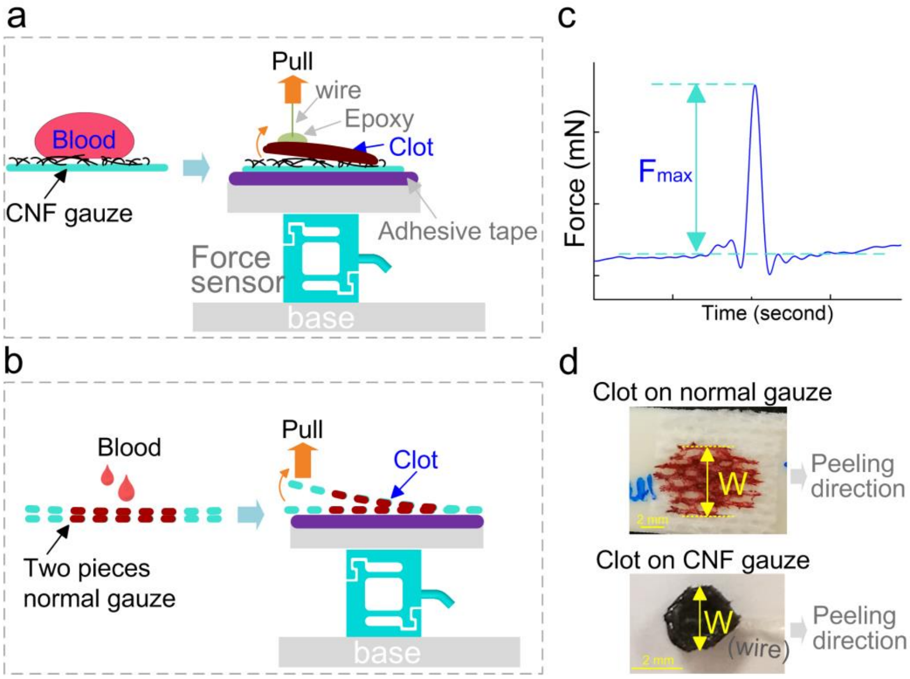

Supplementary Figure 13. Measurement of the clot peeling tension. (a) Clot was formed by dispensing 20 μl blood onto the CNF gauze surface; the gauze was mounted onto a strain gauge force transducer (capacity: 980 mN, resolution: 0.1 mN) by 3M high-strength tape; a thin cotton wire was glued onto the clot by epoxy; the force for peeling the clot was recorded by the force sensor. (b) Clot was formed between two normal gauzes by dispensing 20 μl blood; the force required to peel the two normal gauzes was measured. (c) A typical clot peeling force curve, showing the maximum peeling force F_{max}. (d) Measurement of the clot maximum width W for the calculation of the clot peeling tension F_{max}/W. As the peak peeling force F_{max} would occur at the maximum clot width W, the normalized clot peeling tension F_{max}/W was used to compare the clot peeling force for the CNF gauze and the uncoated normal gauze.

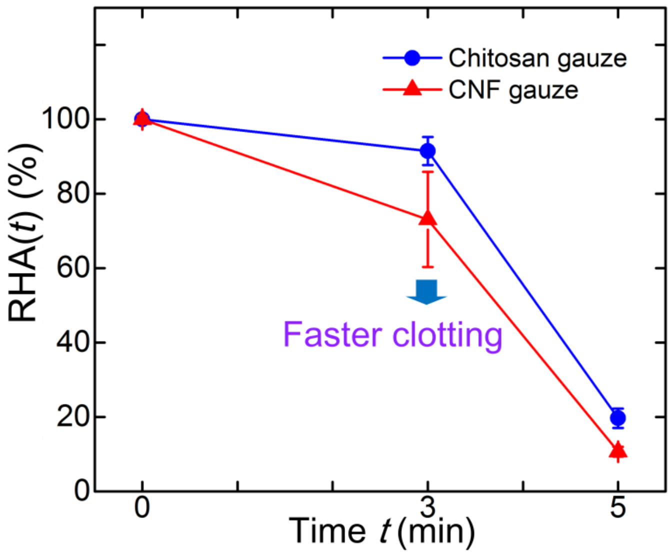

Supplementary Figure 14. The relative hemoglobin absorbance RHA(*t*) plot. Clotting performance of our CNF gauze is compared with the chitosan gauze. The test was performed by sandwiching 50 μl blood between two pieces of CNF gauze or chitosan gauze; data are shown as mean ± s.d., and the error bar represents s.d. (n = 3). A smaller RHA(*t*) means faster clotting. It shows that our CNF gauze can have a better clotting performance than the chitosan gauze.

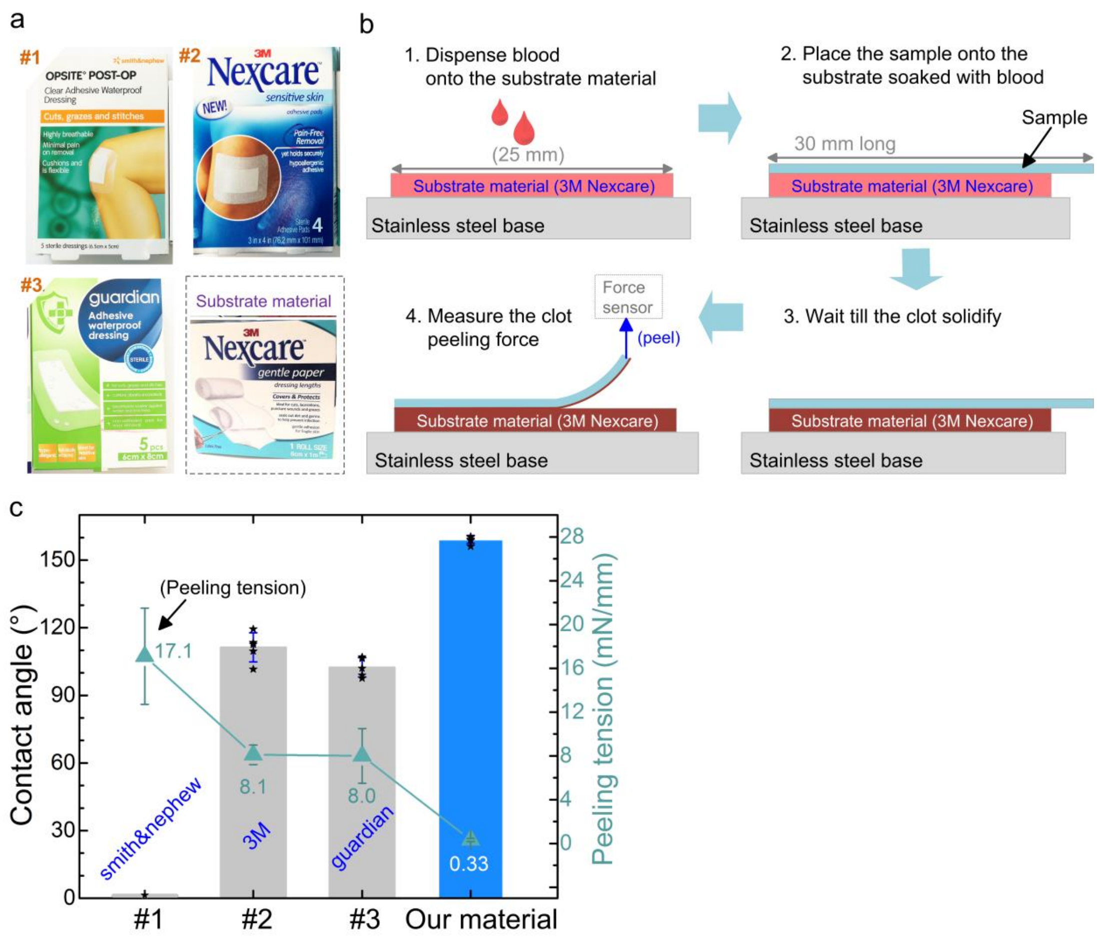

Supplementary Figure 15. Comparison of the clot peeling tension of our material with commercial products. (a) Product #1is the dressing from Smith & Nephew, labeled to have "minimal pain on removal", product #2 is a pad from 3M, labeled to have "pain-free removal", and product #3 is from Guardian, labeled to be a "non-adherent pad for easy removal"; the substrate material is 3M Nexcare. (b) Illustration of the procedures used to measure the clot peeling force: 1) the substrate material was prepared to be 25 mm wide, and adhered onto a stainless steel base, blood was mixed with 0.2 M $CaCl_2$ solution at a volume ratio of 10:1 to initiate coagulation and was immediately dispensed to soak the substrate material; 2) samples prepared to be 30 mm long and 10 mm wide were placed on the substrate; 3) the clot was allowed to form and solidify between the sample and the substrate material; 4) the sample was peeled from one side to measure the peeling force. (c) Summary of the water contact angle and the clot peeling tension for different samples; contact angle was measured with 10 µl DI water (n = 5); peeling tension was calculated as the maximum peeling force divided by the width (repeated for 3 times; n = 3); data are shown as mean ± s.d., s.d. is represented by the error bar, individual data points for the contact angle are represented by black stars.

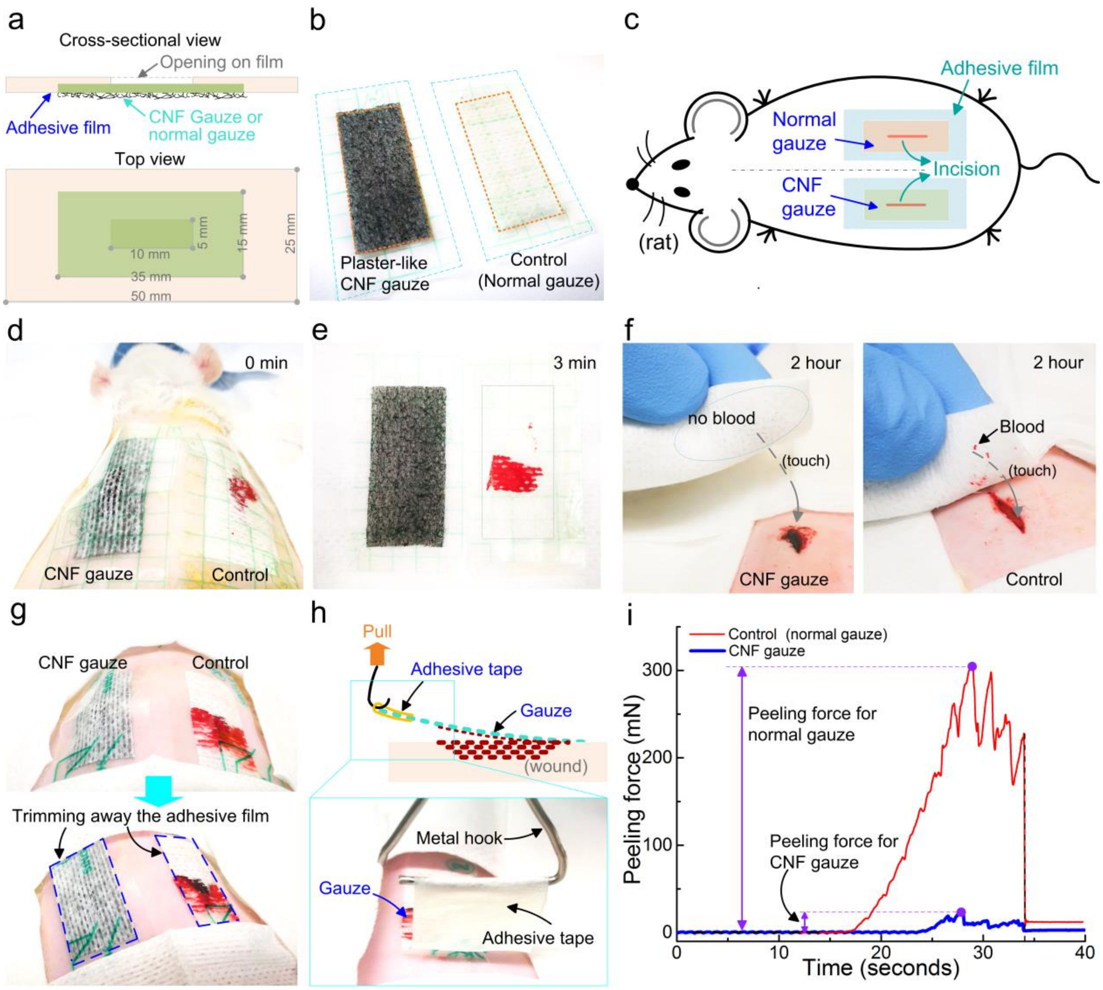

Supplementary Figure 16. *In vivo* experiment. (a) Design of the plaster-like gauze; the CNF gauze or the normal gauze (35 mm by 15 mm) was applied onto the adhesive film (50 mm by 25 mm); an opening of 10 mm by 5 mm was made on the adhesive film, allowing blood to seep through in case of excessive bleeding. (b) The prepared plaster-like CNF gauze and the control normal gauze. (c) Schematic application of gauze onto the wound made on the back of anethesized and shaved rat. (d) The CNF gauze and the control gauze applied onto the wounds on rat back. (e) Comparison between the CNF gauze and the normal gauze peeled at 3 minutes. (f) Peeling the CNF gauze from the wound did not cause secondary bleeding, while peeling the normal gauze torn the wound and caused bleeding (Supplementary Movie 6 and 7). (g) Trimming away the adhesive film around the gauze by scissors as preparation for peeling force measurement, otherwise, the adhesive film taped onto skin would interfere with the peeling force; care was taken not to stretch or tear the gauze during the trimming. (h) Illustration of the peeling force measurement process; after adhesive film trimming, one end of the gauze was taped onto a metal hook by adhesive tape, which was connected onto the force sensor by a thin cotton wire for peeling; peeling the gauze along the wound was assisted by a vertical numerical translation stage. (i) Two typical peeling force versus time curves for the normal gauze and the CNF gauze, and the maximum peeling force was used for comparison (n = 5).

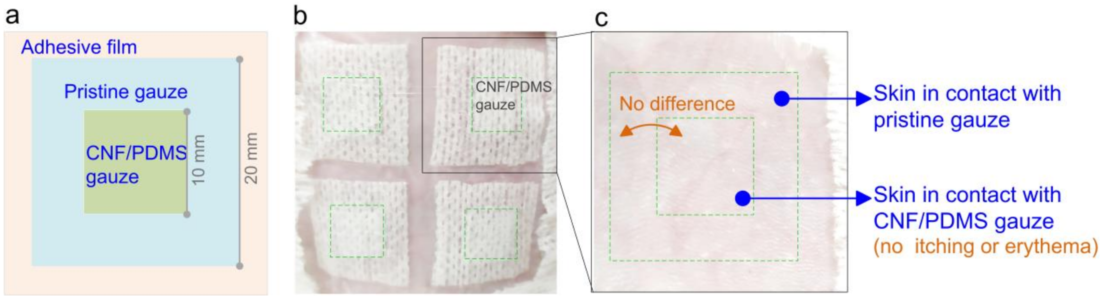

Supplementary Figure 17. *In vivo* skin compatibility test. The test was performed by patching our CNF/PDMS material on rat skin for 12 hours (tested on 3 rats). (a) Schematic illustration of the prepared sample; the CNF/PDMS gauze (10 mm × 10 mm) was attached onto one side of the pristine gauze (20 mm × 20 mm) by the double-sided tape, and a transparent adhesive film was attached onto the other side of the pristine gauze. (b) Four pieces of prepared samples were attached onto rat skin with hairs shaved; the CNF surface was in contact with rat skin. (c) After 12 hours, no difference could be identified between the skin areas in contact the CNF/PDMS gauze and the pristine gauze; the skin in contact with our CNF/PDMS gauze appeared normal and no itching or erythema was observed, showing that our material would be safe for hemostatic use.

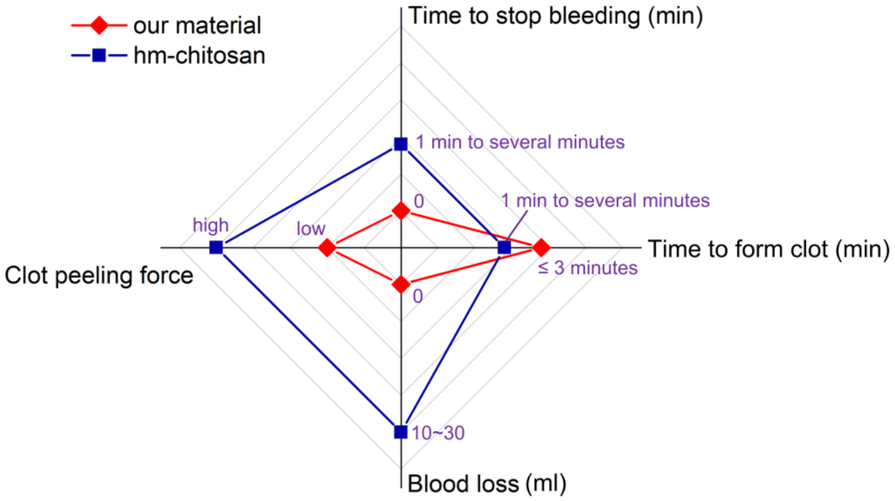

Supplementary Figure 18. Comparison between our material and the hydrophobically modified chitosan[1-3] (hm-chitosan). Comparison was made from 4 important hemostatic performance measures. Compared with hm-chitosan, our material stops bleeding instantaneously upon application, without blood loss; but it takes time for the hm-chitosan to form a blood gel to stop bleeding, with inevitable blood loss (the blood loss was reported to be about 29.3 to 48.9 ml/kg[1] or about 10-30 ml[2]). Regarding the clotting time, our CNF gauze can form a clot within 3 minutes based on our *in vivo* observation (Fig. 5b in the manuscript), while the clotting time for hm-chitosan is affected by its application dose and manner (< 1 min when used in liquid phase[3], > 2 min when used as bandage[3], or "within minutes" when used as a sprayable foam[2]). Thus, hm-chitosan can be more efficient at achieving clotting faster. As for the clot peeling force, our material naturally detached with a very small peeling force and did not adhere on the wound, while the hm-chitosan bandage was reported to adhere on the wound.[3] In summary, hm-chitosan can be faster in forming a clot; our material is superior in reducing the bleeding time, reducing the blood loss, and decreasing the peeling force.

Supplementary References

1 Dowling, M. B. *et al.* Hydrophobically-modified chitosan foam: description and hemostatic efficacy. *J Surg Res.* **193**, 316-323 (2015).

2 Dowling, M. B. *et al.* Sprayable Foams Based on an Amphiphilic Biopolymer for Control of Hemorrhage Without Compression. *ACS Biomaterials Science & Engineering* **1**, 440-447 (2015).

3 Dowling, M. B. *et al.* A self-assembling hydrophobically modified chitosan capable of reversible hemostatic action. *Biomaterials* **32**, 3351-3357 (2011).
