# Supplementary material for: Superhydrophobic hemostatic nanofiber composites for fast clotting and minimal adhesion
Source: Nat Commun. 2019 Dec 5;10:5562. doi: 10.1038/s41467-019-13512-8 (PMC6895059; doi:10.1038/s41467-019-13512-8)
Supplement: Supplementary file 9 — Reporting Summary [file 41467_2019_13512_MOESM9_ESM.pdf]

natureresearch

Corresponding author(s): Choon Hwai Yap, Dimos Poulikakos

Last updated by author(s): Oct 8, 2019

Reporting Summary

Nature Research wishes to improve the reproducibility of the work that we publish. This form provides structure for consistency and transparency in reporting. For further information on Nature Research policies, see Authors & Referees and the Editorial Policy Checklist.

Statistics

For all statistical analyses, confirm that the following items are present in the figure legend, table legend, main text, or Methods section.

n/a	Confirmed	
[ ]	[x]	The exact sample size (*n*) for each experimental group/condition, given as a discrete number and unit of measurement
[ ]	[x]	A statement on whether measurements were taken from distinct samples or whether the same sample was measured repeatedly
[x]	[ ]	The statistical test(s) used AND whether they are one- or two-sided<br>*Only common tests should be described solely by name; describe more complex techniques in the Methods section.*
[x]	[ ]	A description of all covariates tested
[x]	[ ]	A description of any assumptions or corrections, such as tests of normality and adjustment for multiple comparisons
[ ]	[x]	A full description of the statistical parameters including central tendency (e.g. means) or other basic estimates (e.g. regression coefficient) AND variation (e.g. standard deviation) or associated estimates of uncertainty (e.g. confidence intervals)
[x]	[ ]	For null hypothesis testing, the test statistic (e.g. *F*, *t*, *r*) with confidence intervals, effect sizes, degrees of freedom and *P* value noted<br>*Give P values as exact values whenever suitable.*
[x]	[ ]	For Bayesian analysis, information on the choice of priors and Markov chain Monte Carlo settings
[x]	[ ]	For hierarchical and complex designs, identification of the appropriate level for tests and full reporting of outcomes
[x]	[ ]	Estimates of effect sizes (e.g. Cohen's *d*, Pearson's *r*), indicating how they were calculated

Our web collection on statistics for biologists contains articles on many of the points above.

Software and code

Policy information about availability of computer code

Data collection

1. Contact angle: Contact angle in Fig. 1, Fig. 2 and Supplementary Fig. 1a for water and blood on the sample surface was measured using a homemade apparatus (Supplementary Figure 2) with a CCD camera (resolution: 1920x1080 pixels).
2. Roll off angle: Roll off angle in Fig. 1, Fig. 2 and Supplementary Fig. 1a for water and blood on our samples was measured by taking a video (resolution: 1920x1080 pixels; frame rate: 25 fps) while titling the rotational stage until the droplet rolled off the surface.
3. Fibrin data: Fibrin data in Fig. 2a was collected by measuring the optical density (450 nm) with the micro-plate reader.
4. Hemoglobin data: Hemoglobin data in Fig. 3d and Supplementary Fig. 14 was collected by measuring the optical density (540 nm) with the spectrometer.
5. Blood loss: Blood loss in Fig. 3e and Fig. 5d was evaluated by measuring the weight change with a precision weighing balance.
6. Peeling force: The gauze peeling force data in Fig. 4f, Fig. 5e, and Supplementary Fig. 15 was acquired by LabVIEW, using a NI data-acquisition device (NI USB-6218; data sampling rat: 1 kHz).

Data analysis

1. Contact angle analysis: Contact angle was measuring using the software ImageJ (installed with the "contact angle" plugin); after importing images into ImageJ, contact angle was measured using the "Manual Points Procedures" following the instructions of ImageJ.
2. Roll off angle analysis: Roll off angle was measured using the software Tracker; after importing the video into the software, "protractor" (under "Measuring Tools") was used firstly to draw a reference line along the horizontally placed sample surface, and then draw another line along the sample surface at the moment of sudden droplet rolling-off; the angle between these two lines is the roll-off angle.
3-5.The fibrin data, the Hemoglobin data, and the blood loss data were processed with Microsoft Excel.
6. Peeling force analysis: The peeling force was analyzed using the software Origin. Raw force data was imported into Origin, smoothed using the FFT filter (points of window: 50), and the maximum peeling force was then acquired from the force plot.

For manuscripts utilizing custom algorithms or software that are central to the research but not yet described in published literature, software must be made available to editors/reviewers. We strongly encourage code deposition in a community repository (e.g. GitHub). See the Nature Research guidelines for submitting code & software for further information.

Data

Policy information about availability of data

All manuscripts must include a data availability statement. This statement should provide the following information, where applicable:
- Accession codes, unique identifiers, or web links for publicly available datasets
- A list of figures that have associated raw data
- A description of any restrictions on data availability

We have provided a "data availability statement" in the manuscript.
There is no data required for mandatory deposition;
Figures that have associated raw data are: Fig. 2a and d, Fig. 3d and e, Fig. 4f, Fig. 5d and e, Supplementary Fig. 1a, Supplementary Fig. 10b, Supplementary Fig. 14 and 15.

Field-specific reporting

Please select the one below that is the best fit for your research. If you are not sure, read the appropriate sections before making your selection.

[x] Life sciences [ ] Behavioural & social sciences [ ] Ecological, evolutionary & environmental sciences

For a reference copy of the document with all sections, see nature.com/documents/nr-reporting-summary-flat.pdf

Life sciences study design

All studies must disclose on these points even when the disclosure is negative.

Sample size

Sample size for the in vivo rat experiment:
n=6 (6 rats) for measuring in vivo blood loss on rats;
n=5 (5 rats) for measuring the in vivo peeling force;
n=3 (3 rats) for the skin biocompatibility test;

Data exclusions

For either the blood loss, or the peeling force measurement, experiment was firstly performed on the 1 rat for each test to verify the experiment design (this was included in our approved protocol by the university).
Data from the trial group was excluded.

Replication

(1) Blood loss experiment was repeated on 6 different rats;
(2) Peeling force experiment was performed on 5 different rats;
results on each rat follow a general trend: our CNF gauze material has a smaller blood loss and a smaller peeling force than the normal control gauze.
(3) Skin biocompatibility was tested at 4 different locations on each rat.

Randomization

Randomization is not relevant to our study.
In our study, we applied 1 CNF gauze and 1 control gauze, on the same position of rate back, symmetrically one on the left side and one on the right side.

Blinding

There were two investigators for the in vivo rat experiment (Li and Zheng). They performed incisions on rat back, in turn, to minimize human error. The blood loss measurement (by measuring the weight of the gauze before and after the experiment) and the peeling force were acquired by both of them. Data analysis was firstly performed by Li, and subsequently verified by Zheng, re-checked by Yap, and finally confirmed by other co-authors.

Reporting for specific materials, systems and methods

We require information from authors about some types of materials, experimental systems and methods used in many studies. Here, indicate whether each material, system or method listed is relevant to your study. If you are not sure if a list item applies to your research, read the appropriate section before selecting a response.

Materials & experimental systems

n/a	Involved in the study	
[x]	[ ]	Antibodies
[x]	[ ]	Eukaryotic cell lines
[x]	[ ]	Palaeontology
[ ]	[x]	Animals and other organisms
[x]	[ ]	Human research participants
[x]	[ ]	Clinical data

Methods

n/a	Involved in the study	
[x]	[ ]	ChIP-seq
[x]	[ ]	Flow cytometry
[x]	[ ]	MRI-based neuroimaging

Animals and other organisms

Policy information about studies involving animals; ARRIVE guidelines recommended for reporting animal research

Laboratory animals	Rats (Sprague Dawley, female, 11-13 weeks old) were ordered from InVivos Pte Ltd, Singapore, with Institutional approval.
Wild animals	Nil
Field-collected samples	Nil
Ethics oversight	In vivo animal experiment was performed with institutional approval from the National University of Singapore Institutional Animal Care and Use Committee (protocol No. is R18-0961), complying with all relevant ethical regulations for animal testing and research.

Note that full information on the approval of the study protocol must also be provided in the manuscript.
