# Supplementary material for: Superhydrophobic hemostatic nanofiber composites for fast clotting and minimal adhesion
Source: Nat Commun. 2019 Dec 5;10:5562. doi: 10.1038/s41467-019-13512-8 (PMC6895059; doi:10.1038/s41467-019-13512-8)
Supplement: Supplementary file 10 — Description of Additional Supplementary Files [file 41467_2019_13512_MOESM10_ESM.pdf]

Title: Supplementary Movie 1:
Description: On the superhydrophobic CNF/PTFE Ti flat surface, fibrin fibers are forming at the receding side of a 20 µl platelet poor plasma (PPP) droplet with anti-coagulant EDTA. This movie also reveals the importance of proper illumination for visualizing the fibrin fibers at the receding side (at time: 2 seconds, in the Movie). Fibrin fiber footprints are visible on the surface, after catastrophic fiber fracture and droplet rolling down.

Title: Supplementary Movie 2:
Description: On the superhydrophobic CNF/PTFE Ti flat surface, fibrin fibers are forming at the receding side of a 20 µl blood droplet with anti-coagulant EDTA.

Title: Supplementary Movie 3:
Description: On the superhydrophobic CNF/PTFE Ti mesh surface, abundant fibrin fibers are generating at the receding side of a blood droplet with anti-coagulant 3.8% sodium citrate.

Title: Supplementary Movie 4:
Description: On the superhydrophobic CNF/PDMS Ti flat surface, fibrin fibers are forming at the receding side of a 20 µl PPP droplet with anti-coagulant EDTA.

Title: Supplementary Movie 5:
Description: On the superhydrophobic CNF/PDMS Ti flat surface, fibrin fibers are forming at the receding side of a 20 µl blood droplet with anti-coagulant 3.8% sodium citrate.

Title: Supplementary Movie 6:
Description: The superhydrophobic CNF gauze (applied on the wound for about 2 hours) can be easily peeled off, without tearing the wound.

Title: Supplementary Movie 7:
Description: There is a strong adhesion between the normal gauze and the wound. Peeling the normal gauze at about 2 hours seriously tore the wound, and caused secondary bleeding.
